# Correction: Mitochondrial oxidative phosphorylation in peripheral blood mononuclear cells is decreased in chronic HIV and correlates with immune dysregulation

**DOI:** 10.1371/journal.pone.0249428

**Published:** 2021-03-25

**Authors:** Louie Mar A. Gangcuangco, Brooks I. Mitchell, Chathura Siriwardhana, Lindsay B. Kohorn, Glen M. Chew, Scott Bowler, Kalpana J. Kallianpur, Dominic C. Chow, Lishomwa C. Ndhlovu, Mariana Gerschenson, Cecilia M. Shikuma

There is information missing from the Funding statement. The correct Funding statement is as follows: The study was made possible by the funding and clinical research support from National Institute of Health grants R01HL095135 (CMS), U54MD007584, U54MD007601, P20GM113134 (MG), and 1P20GM125526-01A1 (KJK). Details of the grants may be accessed through: https://report.nih.gov/index.aspx. The funders had no role in study design, data collection and analysis, decision to publish, or preparation of the manuscript.

An additional affiliation is missing for the seventh author. Kalpana J. Kallianpur is also affiliated with Center for Translational Research on Aging, Kuakini Medical Center, Honolulu, Hawaii, USA.
